# Abdominal Circumference and Hypotension Incidence in Caesarean Section Under Spinal Anaesthesia

**DOI:** 10.7759/cureus.68851

**Published:** 2024-09-07

**Authors:** Chhaya Suryawanshi, Yukti Roshni

**Affiliations:** 1 Anaesthesiology, Dr. D. Y. Patil Medical College, Hospital and Research Centre, Dr. D. Y. Patil Vidyapeeth, Pune, IND

**Keywords:** abdominal circumference, caesarean section, hypotension, pregnancy, spinal anaesthesia

## Abstract

Introduction: Hypotension is a common side effect during caesarean deliveries under spinal anaesthesia, influenced by factors like obesity, physiological changes and abdominal circumference (AC). This study delves into the intricate relationship between abdominal circumference and hypotension incidence, spinal anaesthesia levels, hemodynamic shifts, and vasopressor requirements in caesarean sections.

Method: A prospective observational study with 232 term pregnant women undergoing caesarean sections was categorized into two groups based on their abdominal circumference, group A (AC < 95 cm) and group B (AC > 109 cm). The study compared hypotension incidence, achieved spinal anaesthesia levels, hemodynamic changes and ephedrine necessity and dosage between the two groups.

Results: Group B with a larger abdominal circumference, exhibited a significantly higher rate of hypotension, more pronounced hemodynamic changes, higher level of spinal anaesthesia and required more doses of ephedrine to manage hypotension compared to group A with a smaller abdominal circumference.

Conclusion: This study highlights the crucial role of abdominal circumference as a predictive factor for hypotension during caesarean sections under spinal anaesthesia. Recognizing this association enables targeted preventive measures, potentially enhancing maternal and fetal outcomes during such procedures. Awareness of this correlation emphasizes the necessity for tailored interventions to mitigate the risk of hypotension, ensuring safer caesarean deliveries under spinal anaesthesia.

## Introduction

Hypotension is a common and potentially serious side effect associated with spinal anaesthesia for caesarean delivery, occurring in up to 80% of cases without preventive measures. Hypotension, defined as a systolic blood pressure of less than 90 mmHg or a mean arterial pressure (MAP) of less than 65 mmHg, can be a significant complication during spinal anaesthesia for caesarean delivery [1]. Significant hypotension is further characterized by a 40% drop in mean arterial pressure from baseline. The physiological changes of pregnancy, such as increased blood volume, decreased systemic vascular resistance, and aorto-caval compression by the gravid uterus, predispose parturients to hypotension during spinal anaesthesia [2]. Additionally, the rapid and extensive ascent of the sensory block level (SBL) can exacerbate hypotension, increasing the risks of maternal discomfort and fetal hypoxia. Increased abdominal circumference and pressure from the gravid uterus elevate intra-abdominal pressure, influencing the upward spread of anaesthetic agents. Term pregnant women often develop varying degrees of intra-abdominal pressure due to their enlarged uteri [3]. Two primary mechanisms have been proposed to explain the rapid decline in mean arterial pressure observed in pregnant women with larger abdominal circumferences. Firstly, the presence of a large uterus can induce aorto-caval compression, diminishing venous return and cardiac output, particularly in the supine position. The abdominal circumference, reflective of uterine size, correlates with heightened intra-abdominal pressure and uterine enlargement, thereby amplifying the likelihood of hypotension after spinal anaesthesia [4]. Second, engorgement of the epidural venous plexus due to compression of the inferior vena cava by a larger uterus can cause a decrease in the volume and pressure of cerebrospinal fluid (CSF) and restriction of the intrathecal space [5]. Consequently, this phenomenon fosters a more cranial diffusion of spinal anaesthesia and sympathectomy, thereby predisposing individuals to hypotension [6,7]. Maternal hypotension can result in adverse effects such as nausea, vomiting, dizziness, and diminished uteroplacental perfusion, potentially endangering fetal well-being. The purpose of this prospective observational study is to investigate the relationship between the incidence of hypotension and abdominal circumference in parturients undergoing caesarean sections performed under spinal anaesthesia. Additionally, the study evaluates the level of spinal anaesthesia, intraoperative hemodynamic fluctuations, and the necessity for vasopressor administration concerning abdominal circumference.

## Materials and methods

This prospective observational study was carried out in the Department of Anaesthesiology, Dr. D. Y. Patil Medical College, Hospital and Research Centre, Pimpri, Pune for a period of one year with six months required for data collection and six months for data analysis and reporting. According to the study conducted by Thomard et al., the mean arterial pressure among the two groups will be 88.25 +/- 11.84 and 92.33 +/- 10.25 [8]. Taking these values with a power of 80% and significance level of 5% the sample size comes out to be 232 (116 in each group).

Two hundred and thirty-two term pregnant women included in this study of ASA (American Society of Anesthesiologists) grade I or II, aged between 20 to 35 years undergoing elective or emergency caesarean section under spinal anaesthesia gave consent for the study. We excluded patients with multiple pregnancies, high-risk pregnancies (placenta previa, abruption placenta, eclampsia and pre-eclampsia), cardiovascular co-morbidities, patients who refused to give consent for spinal anaesthesia, who have an allergy to drugs used in the study and patients with psychological abnormalities.

All the patients were subjected to pre-operative evaluation and relevant laboratory investigations. The patients were divided into two groups based on an abdominal circumference median of 101 cm, according to Thomard et al. [8]. The abdominal circumference of all patients was measured at the umbilical level in a supine position by one anaesthetist. Group A were patients with a smaller abdominal circumference (<95 cm), N=116. Group B were patients with larger abdominal circumference (>109 cm), N = 116.

Ethical considerations

The study was approved by the Institutional Ethics Sub-committee (Research Protocol No. IESC/FP/69/2023, dated September 25, 2023), Dr. D. Y. Patil Medical College, Hospital and Research Centre, Pimpri, Pune. The study was conducted in the Department of Anaesthesiology,​​​​​ Dr. D. Y. Patil Medical College, Hospital and Research Centre, Dr. D. Y. Patil Vidyapeeth, Pimpri, Pune. Informed written consent was obtained from all participants who were briefed about the study's risks, benefits and voluntary nature. Confidentiality of the patient data was maintained throughout the study.

Methods

All patients in the operating room were monitored with automatic non-invasive blood pressure monitoring, three-lead electrocardiogram, and pulse oximetry before the administration of spinal anaesthesia. 0.5% hyperbaric bupivacaine was used to provide spinal anaesthesia in the lateral or sitting position while maintaining aseptic conditions, with the attending anaesthetist adjusting the doses. The treatment was carried out with a 26-gauge Quincke-Babcock spinal needle (BD {Becton, Dickinson and Company}, Eysins, Switzerland), and the pinprick method was employed to determine the degree of spinal anaesthesia. A T4 degree of spinal anaesthesia was achieved by adjusting the operating table. A co-loading approach was employed to infuse 500 mL of crystalloid fluids pre-operatively and the anaesthetist titrated fluids during the perioperative phase. Blood pressure readings, including systolic and diastolic blood pressure, mean arterial pressure (MAP), and heart rate, were obtained non-invasively at baseline and then minutely for ten minutes after spinal anaesthesia was administered. Intravenous ephedrine bolus was injected when required, and the dosage was recorded in order to maintain a mean arterial pressure of at least 65 mmHg. Hypotension was defined in this study as mean arterial pressure less than 65 mmHg or systolic blood pressure less than 90 mmHg. Significant hypotension was defined as mean arterial pressure falling 40% below baseline.

The statistical analysis was carried out using IBM SPSS Statistics for Windows, Version 24 (IBM Corp., Armonk, NY). The chi-square test or Fisher's exact test was used to compare the numerical and percentage representations of the categorical variables. The t-test was used to compare quantitative variables. The relationship between maximal mean arterial pressure change from baseline and abdominal circumference (AC) was investigated using Spearman's rho. Less than 0.05 was the cut-off for statistical significance.

## Results

A total of 232 patients were enrolled in the study and assessed. Group A had patients with smaller abdominal circumference (<95 cm), N=116 and group B had patients with larger abdominal circumference (>109 cm), N=116.

According to our study, the age groups were 27.5 ± 3.2 in group A and 27.7 ± 3.8 in group B and the two groups were comparable without statistical significance (p value= 0.665). The mean weight in group A was 62.4 ± 6.8 and in group B was 89.2 ± 11.5 with statistical significance between the two groups (p<0.001). Body mass index (BMI) was statistically significantly higher in group B 34.8 ± 3.6 as compared to group A 24 ± 2.1 (p-value<0.001) (Table 1).

**Table 1:** Demographic characteristics of the study groups. Data presented as (mean±SD), p-value by t-test and chi-square test.

Variable	(mean ± SD) Group A	(mean ± SD) Group B	p-value
Age (years)	27.5 ± 3.2	27.7 ± 3.8	0.665
Weight (kg)	62.4 ± 6.8	89.2 ± 11.5	<0.001
Height (cms)	161.2 ± 5.1	159.8 ± 6.3	0.064
Body mass index (BMI) (kg/m2)	24 ± 2.1	34.8 ± 3.6	<0.001

We found that immediately after spinal anaesthesia pulse rate increased (p-value<0.001), systolic blood pressure (SBP), mean arterial pressure (MAP) and diastolic blood pressure (DBP) decreased in group B compared to group A (p<0.001). At five minutes after spinal, systolic blood pressure, mean arterial pressure and diastolic blood pressure were statistically low in group B compared to group A (p-value<0.001) whereas pulse rates were comparable between the two study groups. After 10 minutes of spinal, diastolic blood pressure was statistically low in group B when compared to group A (p<0.001) whereas systolic blood pressure, mean arterial pressure and pulse rate were comparable and had no statistical significance in both the study groups (Table 2).

**Table 2:** Hemodynamic parameters (pulse, systolic blood pressure, diastolic blood pressure, mean arterial pressure) are compared between the two study groups at different time intervals. Data presented as (mean ± SD), p-value by t-test and chi-square test.

Variables	Time	(mean ± SD) Group A	(mean ± SD) Group B	p-value
Pulse	Baseline	84.2 ± 7.1	85.4 ± 8.5	0.244
	Immediately after spinal	86.4 ± 8.3	92.6 ± 9.7	<0.001
	5 minutes after spinal	90.2 ± 8.9	91.8 ± 10.2	0.204
	10 minutes after spinal	88.6 ± 8.6	90.4 ± 10.8	0.162
Systolic blood pressure (SBP)	Baseline	116.2 ± 8.4	117.6 ± 8.1	0.198
	Immediately after spinal	98.4 ± 10.2	82.8 ± 12.6	<0.001
	5 minutes after spinal	96.6 ± 11.8	86.4 ± 14.3	<0.001
	10 minutes after spinal	100.8 ± 10.6	98.2 ± 13.7	0.107
Diastolic blood pressure (DBP)	Baseline	80.2 ± 8.4	78.6 ± 9.1	0.165
	Immediately after spinal	68.4 ± 10.2	48.8 ± 12.6	<0.001
	5 minutes after spinal	60.6 ± 11.8	50.4 ± 14.3	<0.001
	10 minutes after spinal	65.8 ± 10.6	50.2 ± 13.7	<0.001
Mean arterial pressure (MAP)	Baseline	92.5 ± 5.8	93.6 ± 6.2	0.164
	Immediately after spinal	65.7 ± 8.2	62.7± 9.8	<0.001
	5 minutes after spinal	63.6 ± 9.4	56.1 ± 11.2	<0.001
	10 minutes after spinal	64.1 ± 8.6	63.1 ± 10.6	0.431

Sixty-eight patients (58.6%) in group B had hypotension when compared to 28 (24.1%) in group A and the difference was statistically significant in both groups (p<0.001). The requirement of ephedrine was more in group B 9.8 ± 4.6 compared to group A 4.2 ± 3.1 and there was statistical significance in both groups (p<0.001) (Table 3).

**Table 3:** Comparison of outcome variables (hypotension) and requirement of ephedrine among study groups. Data presented as (mean±SD), p-value by t-test and chi-square test. IQR: Interquartile range.

Outcome variables	(mean ± SD) Group A	(mean ± SD) Group B	p-value
Hypotension (n, %)	28 (24.1%)	68 (58.6%)	<0.001
Significant hypotension (n, %)	12 (10.3%)	38 (32.8%)	<0.001
Level of spinal anaesthesia (median, IQR)	T6 (T4-T8)	T4 (T3-T6)	-
Ephedrine required (n, %)	40 (34.5%)	92 (79.3%)	<0.001
Ephedrine dose (mg)	4.2 ± 3.1	9.8 ± 4.6	<0.001

## Discussion

Our findings demonstrated a higher incidence of hypotension with a significant fall in baseline systolic blood pressure (SBP), diastolic blood pressure (DBP) and mean arterial pressure (MAP) immediately after spinal anaesthesia in group B with larger abdominal circumference (>109 cm) {58.6%} compared to group A with smaller abdominal circumference (<95 cm) {24.1%} (p<0.001). Compression of the inferior vena cava due to a larger uterus might produce engorgement of the epidural venous plexus. This constricts the intrathecal space and lowers the volume of cerebrospinal fluid (CSF), which may cause the anaesthetic medication to distribute more cephalad and cause hypotension. Additionally, the large uterus can cause aorto-caval compression, which decreases cardiac output and venous return when the patient is in the supine position, thereby increasing the incidence of hypotension. Thomard et al. conducted a similar study, which had a significantly higher incidence of hypotension with a baseline decrease in mean arterial pressure in the group with larger abdominal circumference (62.9%) compared to those with smaller abdominal circumference which is less than or equal to 101 cm (40%) [8]. Patients with smaller abdominal circumferences (group A) had a median level of spinal anaesthesia as T6 (T6-T8) and those with larger abdominal circumferences (group B) had a T4 level (T3-T6). The optimization of spinal drug dosage depending on the patient's body mass index (BMI), height and abdominal circumference plays a pivotal role in preventing the incidence of hypotension. An observational study by Kuok et al. discovered a relationship between the degree of the sensory block during spinal anaesthesia and abdominal circumference [9]. Their findings showed that five minutes after spinal anaesthesia was administered, parturients with higher abdominal circumference showed a higher degree of sensory blockade. The study did find, however, that abdominal circumference was unable to predict the occurrence of hypotension or the maximal sensory blockage level on its own. Abdominal pressure also has an impact on the degree of spinal anaesthesia. It has been demonstrated that higher spinal anaesthesia levels and a higher risk of hypotension are caused by elevated stomach pressure. It is crucial to remember that our study did not include an intra-abdominal pressure measurement. Immediately after spinal anaesthesia pulse rate increased, and systolic blood pressure, mean arterial pressure and diastolic blood pressure decreased in larger abdominal circumference patients compared to smaller abdominal circumference patients (p<0.001). At five minutes after spinal, systolic blood pressure, mean arterial pressure and diastolic blood pressure were statistically low in larger abdominal circumference compared to smaller abdominal circumference. After 10 minutes of spinal diastolic blood pressure was statistically low in larger abdominal circumference when compared to smaller abdominal circumference (p<0.001) whereas the systolic blood pressure, mean arterial pressure and pulse rate were maintained and had not much statistical significance in both the groups. The choice of positioning, such as left lateral tilt or manual displacement of the uterus after spinal anaesthesia can alleviate the aortocaval compression thereby enhancing venous return and cardiac output and helping to maintain stable hemodynamic parameters. According to Thomard et al., there was no discernible variation in heart rate across the groups (p = 0.94) [8]. The larger abdominal circumference group did, however, experience a greater reduction in mean arterial pressure after baseline spinal anaesthesia (MAP at T0-lowest MAP at any point in time between T1 and T10). Ephedrine, a sympathomimetic, increases cardiac output and peripheral resistance, effectively counteracting the hypotensive effects. It is often preferred in obstetric settings because it does not significantly decrease uteroplacental blood flow, ensuring adequate oxygen delivery to the fetus. Ephedrine requirement was significantly higher in the larger abdominal circumference group B (79.3%) compared to the smaller abdominal circumference group A (34.5%) (p<0.001) in our study. According to research by Can et al. patients who had subarachnoid blocks used for caesarean sections showed a strong relationship with their ephedrine use and their abdomen circumference and AC-to-hip ratio [10]. Out of the 60 participants, 40 patients (66.6%) had hypotension that needed to be treated with ephedrine. The amount of ephedrine consumed (mg) and abdominal circumference had a positive link (r=0.280, p=0.03). Ephedrine can be used along with crystalloids or colloids in mitigating hypotension while ensuring maternal and fetal well-being in caesarean sections under spinal anaesthesia.

Limitations

In our study, we excluded patients belonging to the high-risk category and those having allergies to spinal drugs and local anaesthetics. We also excluded patients who underwent caesarean section under general anaesthesia with larger abdominal circumferences and those in whom spinal action failed and had to be converted to general anaesthesia.

## Conclusions

These results emphasize the importance of recognizing abdominal circumference as a significant predictive factor for identifying patients at risk of hypotension following spinal anaesthesia. This awareness can lead to the implementation of targeted preventive measures to enhance maternal and fetal outcomes during caesarean deliveries under spinal anaesthesia. Further research on interventions tailored to variations in abdominal circumference could provide valuable insights into refining perioperative management strategies and improving obstetric anaesthesia care.
